# Picosecond time-resolved infra-red spectroscopic study of a water-soluble cationic copper-porphyrin with nucleic acids

**DOI:** 10.1039/d5cp04939c

**Published:** 2026-03-05

**Authors:** Páraic M. Keane, Daniel Graczyk, Igor V. Sazanovich, Michael Towrie, Susan J. Quinn, John M. Kelly

**Affiliations:** a School of Chemistry, Trinity College Dublin Dublin 2 D02 P3X2 Ireland jmkelly@tcd.ie keanepa@tcd.ie; b School of Chemistry, University of Reading. Whiteknights Reading RG6 6AD UK; c School of Chemistry, University College Dublin Dublin Ireland susan.quinn@ucd.ie; d STFC Central Laser Facility, Research Complex at Harwell, Rutherford Appleton Laboratory Didcot OX11 0QX UK

## Abstract

The greater abundance and lower cost of copper make its porphyrin derivatives an attractive alternative to precious-metal based photosensitisers. The Cu(ii) complex of 5,10,15,20-*meso*-tetrakis(*N*-methylpyridinium-4-yl)porphyrin (CuTMPyP4) is a useful photophysical probe for biomolecules. It is non-luminescent in aqueous solution, as it forms a metastable five-coordinate water complex after photo-excitation. CuTMPyP4 is known to bind to DNA both through the grooves and by intercalation. In this paper picosecond transient absorption (TA) and time-resolved infra-red (TRIR) spectra of CuTMPyP4 in D_2_O and in the presence of polydeoxythymidylic acid (poly(dT) and the double-stranded oligonucleotides {d(GC)_5_}_2_ and {d(CGCAAATTTGCG)}_2_ are reported. These spectra show characteristic features depending on whether a five-coordinate transient species or the four-coordinate triplet excited state is formed. Notably, in the case of thymine-containing nucleic acids the TRIR method unambiguously demonstrates the binding of the porphyrin to the C_2_=O carbonyl group of that nucleobase.

## Abstract 2

TRIR spectra show distinctive features when CuTMPyP4 forms a five-coordinate ‘exciplex’ species, *e.g.* in D_2_O or with thymine in poly(dT), or the triplet excited state in {d(GC)_5_}_2_. They show binding to the C2=O of thymine in {d(CGCAAATTTGCG)}_2_.

## Graphical abstract

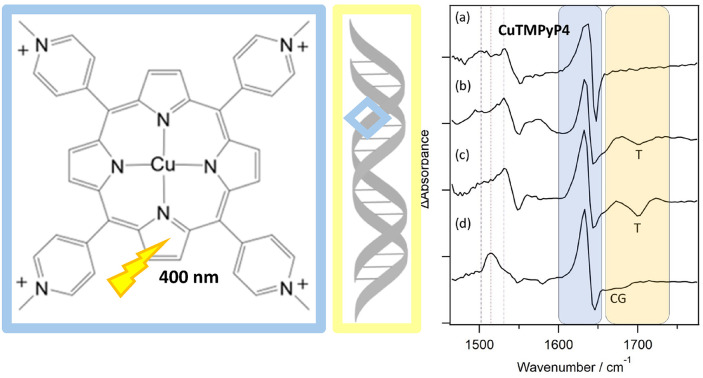

## Introduction

Porphyrin molecules are an important class of photosensitisers for applications ranging from artificial photosynthesis to photodynamic therapy.^1^ The binding of *meso*-tetrakis(*N*-methylpyridinium-4-yl) porphyrins to nucleic acids has been a rich field of study for many years.^2^ As binding to DNA perturbs the excited states of the porphyrins, optical spectroscopic methods have been widely used to study the nature of the binding processes for both the free base ligand (H_2_TMPyP4) and its metallo-derivatives (MTMPyP4). Emission spectroscopy is particularly valuable as the luminescence is sensitive not only to the mode of binding (intercalation, groove binding *etc.*) but also to the base sequence. For example, H_2_TMPyP4 fluorescence is enhanced upon binding to AT-rich sequences in DNA but quenched when intercalated between GC base-pairs.^2,3^ Amongst the metallo-derivatives the fluorescence of ZnTMPyP4 is enhanced upon DNA-binding^2^ as is the phosphorescence of PtTMPyP4.^4^

**Fig. 1:**
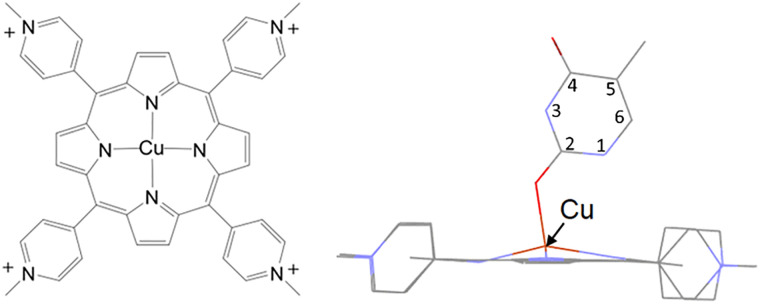
Structure of CuTMPyP4 and calculated structure of CuTMPyP4-(κ-*O*-thymine) reproduced from McGarry *et al.*^9^

The greater abundance and lower cost of copper make their porphyrin derivatives an attractive alternative to precious-metal based photosensitisers. An interesting feature of CuTMPyP4 is that it is non-luminescent in water.^5^ This is attributed to sub-picosecond formation of a 5-coordinate water complex, CuTMPyP4-(κ-*O*-H_2_O), which reforms the ground state CuTMPyP4 in a few picoseconds.^6–9^ This high energy species is called an ‘exciplex’ in the literature. However, recent quantum mechanical calculations indicate that it exists in its electronic ground state, and have shown that the formation of this 5-coordinate species occurs *via* a singlet LMCT excited state.^9^ Evidence for the formation of this high energy 5-coordinate species has come from Raman studies.^6^ The biological activity of copper porphyrins is of significant interest.^10^ CuTMPyP4 is a useful photophysical DNA probe and several studies have explored its binding to different DNA conformations including single, double, left-handed Z-DNA and quadruplex DNA.^11^ CuTMPyP4 is versatile and can bind through multiple modes including end stacking, intercalation, groove binding and electrostatic binding. Notably, studies by the Chirvony, Turpin and Kim groups demonstrated that CuTMPyP4 formed a relatively long-lived (2–3 ns) ‘exciplex’ with thymine-containing nucleic acids through coordination to a carbonyl group of the nucleobase, see Fig. 1.^12–20^

In the current paper we present a picosecond time-resolved visible absorption (TA) and infra-red (TRIR) study of CuTMPyP4 in aqueous solution and in the presence of double-stranded DNAs and with polydeoxythymidylic acid.

In previous studies of DNA-bound polypyridyl metal complexes^21^ or porphyrins^22^ it has been shown that the TRIR technique can simultaneously detect changes in the photosensitiser and the nucleic acid-centred vibrational modes, the latter of which has been termed a ‘site effect’ as these changes arise only in the nucleobases in the immediate environment of the photosensitiser. By comparison, access to information on both the photosensitiser and the binding site is more difficult to achieve using time-resolved resonance Raman techniques. In this paper we demonstrate how the site effect can be exploited to provide information on both the nature of the excited state and the binding site of the CuTMPyP4 to single-stranded and double-stranded nucleic acids.

## Results and discussion

### TA and TRIR investigations of CuTMPyP4 in D_2_O

**Fig. 2:**
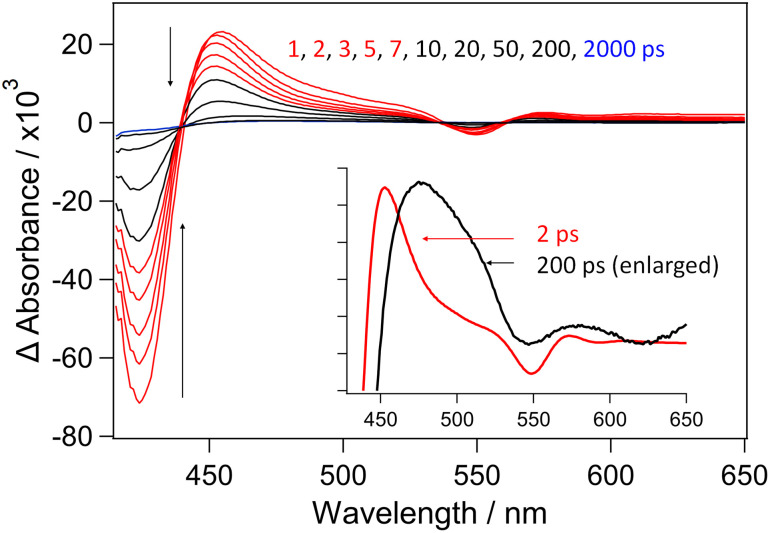
Picosecond TA spectra recorded following 400 nm excitation of 500 µM CuTMPyP4 in buffered D_2_O showing the decay of the 5-coordinate CuTMPyP4-(κ-*O*-D_2_O) and recovery of the four-coordinate CuTMPyP4 in less than 50 ps. Insert: comparison of 2 ps (red) and scaled 200 ps (black) spectra.

The UV/vis spectrum of CuTMPyP4 consists of a strong B-band (Soret) at 424 nm (SI Fig. S1), with a Q-band at 548 nm and another weak Q-band at approx 600 nm.^23^ The TA spectra of CuTMPyP4 in phosphate buffered D_2_O are shown in Fig. 2. As with the corresponding spectra reported in H_2_O solution,^6–9^ these are dominated by the negative (bleach) band (*λ*_max_ 424 nm) due to the removal of the B-band, while the transient band (*λ*_max_ 454 nm) may be assigned to the ‘exciplex’ CuTMPyP4-(κ-*O*-D_2_O). The blue shift of the absorption maximum during the first 5 ps may be attributed to the exciplex being formed in a vibrationally hot state.^7^ This transient decays away over 50 ps following complex kinetics, (see Fig. S2 and Table S1) similar to that previously reported in H_2_O.^6,7^ A weak absorption remains on timescales > 50 ps. This feature has a markedly different spectral profile to that observed at early times (see insert in Fig. 2), and we assign this to the triplet excited state.

**Fig. 3:**
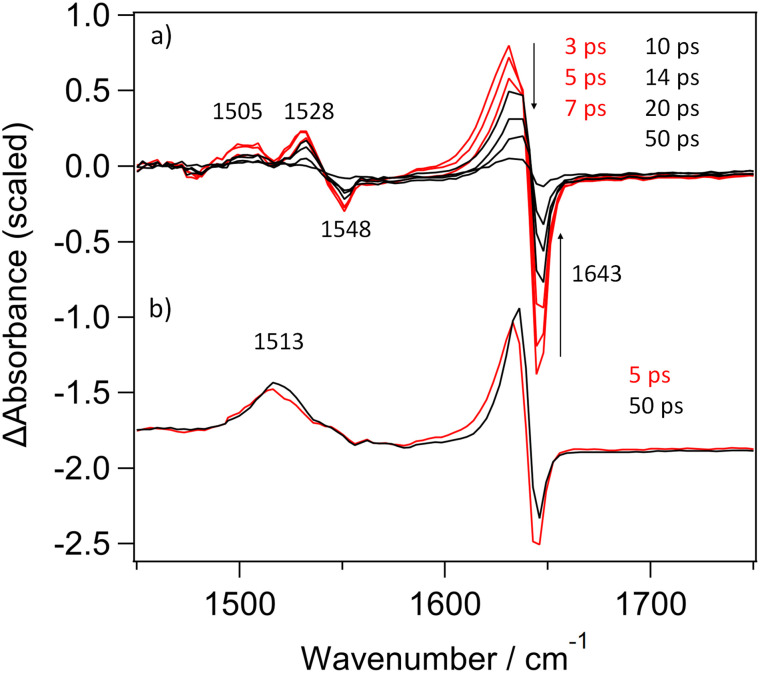
TRIR spectra recorded at various times after 400 nm excitation of (a) 500 µM CuTMPyP4 in buffered D_2_O, (b) PtTMPyP in buffered D_2_O^22^ – scaled to equal intensity of the principal transient band.

The spectroscopic changes in the mid-infra-red region in the picosecond range measured following 400 nm (1 µJ, 120 fs) excitation of CuTMPyP4 in phosphate-buffered D_2_O are presented in Fig. 3a. These show depletion of the parent features at 1643 and 1548 cm^−1^, and formation of product bands at 1632, 1528 and 1505 cm^−1^. The strong negative signal evident at 1643 cm^−1^ is similar to that reported for PtTMPyP4^22^ (see Fig. 3b) and corresponds to the bleach of the pyridinium-centred absorption of CuTMPyP4. The strong absorption observed at 1632 cm^−1^ is associated with the pyridinium modes of the five-coordinate CuTMPyP4-(κ-*O*-D_2_O)-complex. This band is noted to shift from an initial peak at approx. 1625 cm^−1^ over the first 10 ps, consistent with vibrational relaxation. The areas of the positive band centred at 1632 cm^−1^ and that of the bleach signal centred at 1643 cm^−1^ are approximately equal. (By contrast the corresponding ratios for the triplet state of PtTMPyP4 is *ca.* 4 : 1, see Fig. 3b).^22^ The transient monitored at 1632 cm^−1^ can be fitted to biexponential kinetics with lifetimes of 11.2 ± 0.7 ps (88%) and 119 ± 9 (12%), see SI Fig. S3. There is additionally a low-intensity long-lived broad absorption signal centred around 1500 cm^−1^. This probably represents a minor yield of the lowest energy triplet state of CuTMPyP4 (SI Fig. S4), analogous to what was observed in the TA spectra (Fig. 2).

### TA and TRIR investigations of CuTMPyP4 with {d(GC)_5_}_2_ in D_2_O

We next studied CuTMPyP4 in the presence of the double-stranded oligo-deoxynucleotide {d(GC)_5_}_2_, where the porphyrin is expected to be intercalated between the GC base-pairs.^24^ This was confirmed by characteristic hypochromism and red-shift of the CuTMPyP4 B (Soret) band in the UV/vis on binding to {d(GC)_5_}_2_. In contrast to the unbound CuTMPyP4, intercalation between GC base-pairs results in a weak luminescence at 770 nm (SI Fig. S5), which is indicative of formation of the emissive triplet state.^5^

**Fig. 4:**
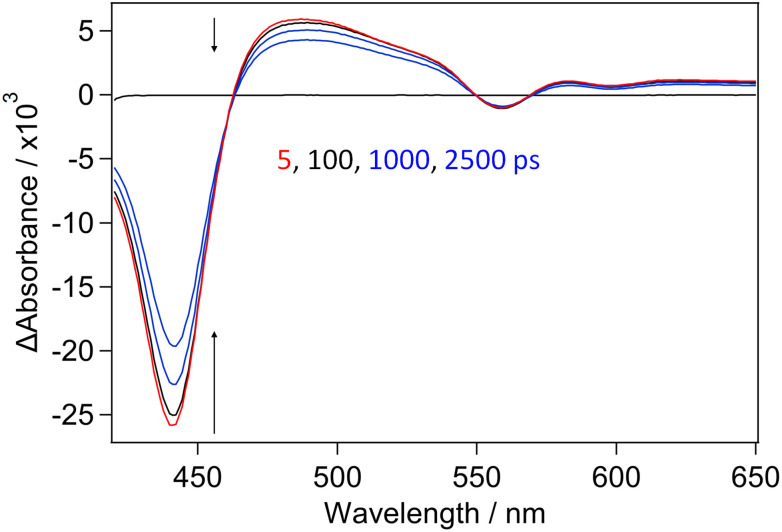
ps-TA spectra of CuTMPyP4 (500 µM) in the presence of {d(GC)_5_}_2_ (500 µM, CuTMPyP4 : Nucl = 1 : 20) in buffered D_2_O solution following 400 nm excitation. Delays shown at 5, 100, 1000 and 2500 ps.

The transient visible absorption changes (Fig. 4) produced for CuTMPyP4 bound to {d(GC)_5_}_2_, are quite different to those observed in D_2_O. The absorption of the transient maximises at 487 nm and the band is much broader than that recorded in pure buffered D_2_O. This transient species is also much longer-lived with a lifetime greater than 1 ns. This is consistent with the species observed being the triplet state, which is known to decay on the ns timescale (2.8 ns, 61%; 22 ns, 39% in {poly(dG-dC)}_2_).^15^ It may be noted that as the CuTMPyP4 is intercalated between the GC base-pairs, access by water to the copper atom is hindered and hence formation of the CuTMPyP4-(κ-*O*-D_2_O) complex is inhibited.

**Fig. 5:**
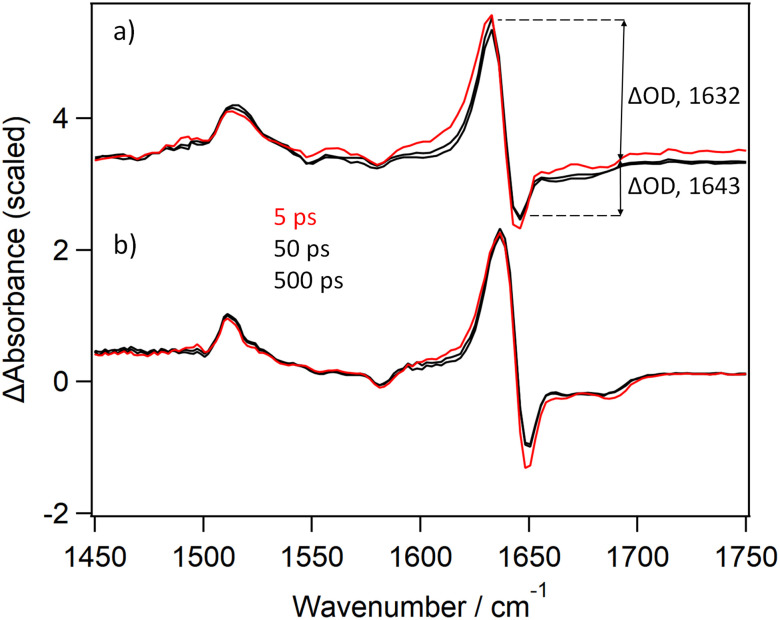
TRIR spectra of (a) CuTMPyP4 (500 µM) in the presence of {d(GC)_5_}_2_ (500 µM, CuTMPyP4 : Nucl = 1 : 20) and (b) PtTMPyP4 (500 µM) in the presence of {d(GC)_5_}_2_ (500 µM, CuTMPyP4 : Nucl = 1 : 20). Both in D_2_O buffer with 400 nm excitation (1 µJ). For ease of comparison absorbance has been scaled for matching intensity of 1632 cm^−1^ transient. Measurement of the ratios of band intensities at 1632 cm^−1^ and 1643 cm^−1^ at 50 ps (ΔAbs._(1632/1643)_) is depicted in (a).

The TRIR of CuTMPyP4 in the presence of {d(GC)_5_}_2_ is dominated by strong absorption bands at 1513 and 1632 cm^−1^ (Fig. 5a). The spectrum observed, which we assign to the CuTMPyP4 triplet state, is similar to that observed for the triplet state of PtTMPyP4 intercalated into {d(GC)_5_}_2_ (Fig. 5b).^22^ Thus, it shows the same band at 1513 cm^−1^, and enhanced pyridinium absorption band (at 1632 cm^−1^) relative to the CuTMPyP4 bleach at 1643 cm^−1^ (ΔAbs._(1632/1643)_ = 3.2 at 50 ps), see Fig. 5a.

Additional long-lived bleach signals are also apparent at 1580 and 1682 cm^−1^. These coincide with those of the ring and C=O vibrations of guanine^25^ (see also Fig. S6), respectively, and are consistent with the perturbation of these guanine-centred vibrations by proximity to CuTMPyP4 in its lowest energy triplet state. Similar effects have been observed with PtTMPyP4^22^ and ruthenium polypyridyls.^21^

### TRIR investigations of CuTMPyP4 with polydeoxythymidylic acid in D_2_O solution

**Fig. 6:**
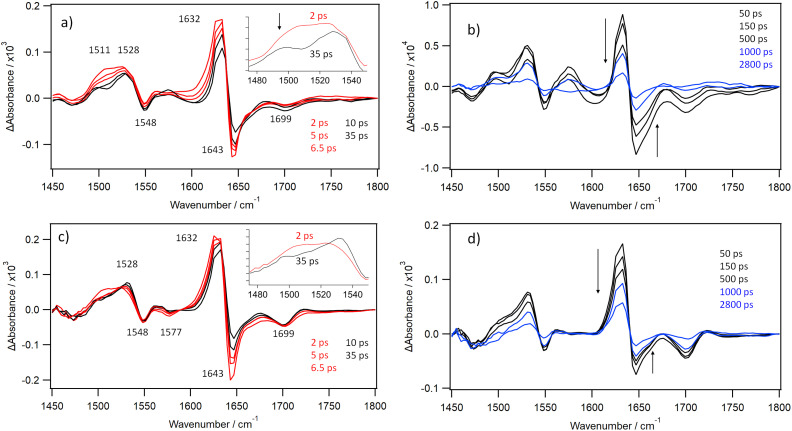
(a) The early-time and (b) later time TRIR spectra obtained following 373 nm excitation of CuTMPyP4 (500 µM) in the presence of poly(dT) (10 mM nucleotide, CuTMPyP4 : Nucl = 1 : 20) in buffered D_2_O (c) The early time TRIR spectroscopic changes observed following 373 nm excitation of 0.5 mM CuTMPyP4 in the presence of {d(CGCAAATTTGCG)}_2_ in D_2_O (1.0 mM per strand, CuTMPyP4 : Nucl = 1 : 24) showing the depletion of the CuTMPyP4 bands at 1643 cm^−1^ and 1548 cm^−1^, features characteristic of loss of ground state CuTMPyP4 and a significant depletion at 1699 cm^−1^ corresponding to the bleaching of a uncoordinated thymine absorption; (d) the later time changes showing the slow decay of all product bands and recovery of the parent absorptions.

**Fig. 7:**
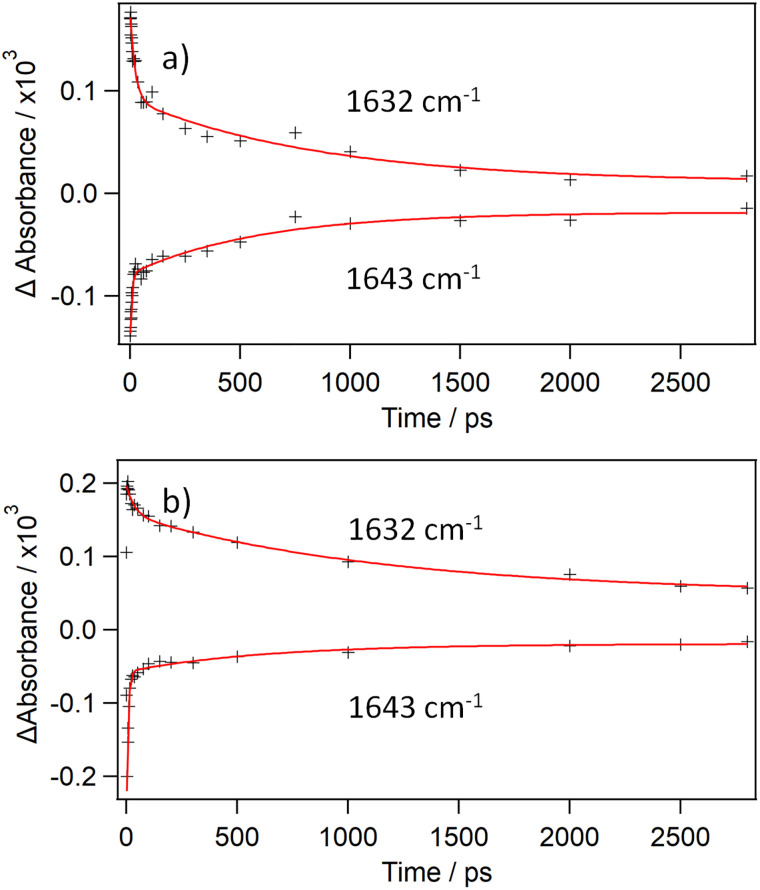
Biexponential TRIR kinetic fits of CuTMPyP4 (500 µM) in the presence of (a) poly(dT) (10 mM nucleotide, CuTMPyP4 : Nucl = 1 : 20) (b) d(CGCAAATTTGCG)}2 (1.0 mM per strand, CuTMPyP4 : Nucl = 1 : 24). Both in 50 mM phosphate buffered D_2_O.

The TRIR spectroscopic changes observed following 373 nm excitation of CuTMPyP4 in solution with polydeoxythymidylic acid (poly(dT)) in buffered D_2_O are presented in Fig. 6(a) and (b). The signals in the 1620–1650 cm^−1^ region clearly show biexponential behaviour (Fig. 7a). The product band is noted to shift from 1625 cm^−1^ to 1632 cm^−1^ in the first 20 ps. In the 1500 to 1540 cm^−1^ region there are initially two overlapping components, with maxima at approx. 1511 cm^−1^ and 1528 cm^−1^. The 1511 cm^−1^ absorption then diminishes during the first 20 ps, while the absorbance at 1528 cm^−1^ is comparatively unchanged over the first 20 ps. The loss of the band at 1511 cm^−1^ reveals the distinct absorption at 1500 cm^−1^ associated with the 1528 cm^−1^ feature, see inset of Fig. 6a. It may also be noted that in poly(dT) the ratio of the 1632 cm^−1^ and 1643 cm^−1^ bands (ΔAbs._(1632/1643)_) for the long-lived species is 1.4, which is quite different from what is found when CuTMPyP4 is bound to {d(GC)_5_}_2_ (ratio = 3.2) and more similar to what is observed in the absence of polynucleotide. A comparison between CuTMPyP4 in D_2_O and poly(dT) is given in SI Fig. S7. An additional feature in the poly(dT) system is a bleach band at 1699 cm^−1^ which is expected for the C2=O carbonyl absorption of thymine. We therefore assign this long-lived species (*τ* = 850 ± 280 ps) as the 5-coordinate CuTMPyP4-(κ-*O*-thymine). By way of comparison, using visible transient absorption methods, Chirvony *et al.* measured a lifetime of 950 ps for CuTMPyP4 bound to the short oligonucleotide d(*T*)_*n*_ (*n* = 1–18) in H_2_O solution.^12^ The short-lived species may be CuTMPyP4-(κ-*O*-D_2_O), formed by the porphyrin species still able to access the solvent, or due to an interaction with the second thymine carbonyl (C4=O).

### TRIR investigations of CuTMPyP4 with {d(CGCAAATTTGCG)}_2_ in D_2_O solution

We next investigated the TRIR of CuTMPyP4 in the presence of the mixed sequence double-stranded oligodeoxynucleotide {d(CGCAAATTTGCG)}_2_. This model self-complementary sequence was chosen as it has been the subject of several DNA binding studies and detailed knowledge of its B-DNA structure^26,27^ and of minor groove bound drug molecules^28^ have been revealed by X-ray crystallography. Previous steady-state studies have shown that in mixed sequence DNA CuTMPyP4 may bind both in the minor groove (in the AT-rich section) or intercalate (at a GC base-pair).^29^

The TRIR data following 373 nm excitation are presented in Fig. 6(c) and (d). (essentially similar results were obtained with 400 nm excitation; SI Fig. S8). These data show many similarities to what was observed with CuTMPyP4 bound to poly(dT). The characteristic porphyrin features at 1632 cm^−1^ and 1643 cm^−1^ show biexponential behaviour and a blue-shifting of the main absorption band from 1625 cm^−1^ to 1632 cm^−1^ in the first 20 ps. Additionally, there is initially a broad transient in the 1500–1540 cm^−1^ region which yields a long-lived species with a maximum at 1528 cm^−1^. Other bleach bands occur at 1548 cm^−1^ and 1699 cm^−1^. The latter is caused by the C2=O carbonyl absorption of thymine and might be expected by the formation of the 5-coordinate CuTMPyP4-(κ-*O*-thymine). This long-lived species decays with a lifetime of 1180 ± 340 ps (measured at 1632 cm^−1^, Fig. 7b).

**Fig. 8:**
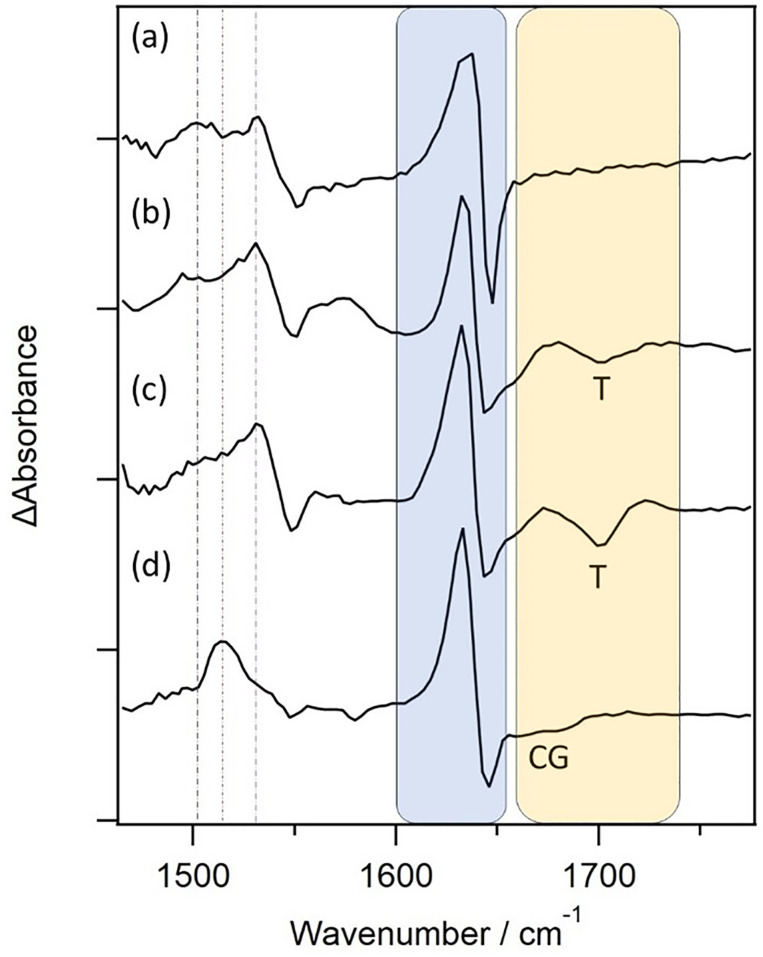
Comparative spectra; (a) 20 ps TRIR spectrum of CuTMPyP4 in D_2_O. 50 ps TRIR spectra of 500 µM CuTMPyP4 in presence of (b) poly(dT) (c) {d(CGCAAATTTGCG)}_2_ and (d) {d(GC)_5_}_2_ Vertical lines track bands associated with 5-coordinate species (1505 cm^−1^, 1528 cm^−1^) and triplet excited state (1513 cm^−1^). Shaded areas denote regions associated with porphyrin pyridinium (blue) and DNA nucleotide carbonyls (yellow).

However, there are some features that differ between the two systems. In contrast to what was observed for the long-lived species in the CuTMPyP4-poly(dT) system, the ΔAbsorbance observed at 1632 cm^−1^ is much larger than that of the bleaching at 1643 cm^−1^ (ΔAbs._(1632/1643)_ = 2.2 for {d(CGCAAATTTGCG)}_2_; 1.4 for poly(dT)), and may also be compared to the ratio observed for CuTMPyP4 bound to {d(GC)_5_}_2_ (3.2), which was assigned to the triplet state (see also Fig. 8). In addition, while the intensity of the signal associated with binding to thymine (1699 cm^−1^) does not change appreciably over the first 20 ps, a weak bleach at 1577 cm^−1^ (expected for a guanine ring vibration as shown above with {d(GC)_5_}_2_) essentially disappears over this time period (SI Fig. S9). The short-lived nature of this process also contrasts with that found when the porphyrin intercalates between the base-pairs of {d(GC)_5_}_2_) and it could involve coordination to the endocyclic nitrogen atoms on the guanine, giving (CuTMPyP4-(κ-*N*-guanine)). This species would be expected to be short-lived, as it should behave similarly to the pyridine 5-coordinate complex recently examined by DFT.^9^

A further notable feature in the TRIR is a weak transient band at 1720 cm^−1^, that emerges after approx. 10 ps and appears relatively long-lived. IR bands above 1700 cm^−1^ in nucleobases are often associated with carbonyls in non-aqueous environments (*e.g.*, over 40 cm^−1^ difference for some thymine derivatives in pure CD_3_CN *vs*. D_2_O).^30^ The high-resolution X-ray structure of {d(CGCAAATTTGCG)}_2_ shows that the A_3_T_3_ minor groove contains a monolayer of water molecules, which bridges the two strands. This can either involve two O4′ atom(s) and one O2 (T) atom and one N3 (A) atom or in the case of the ApT step, two O2 (T) atoms.^27^ Since the disruption appears to exclusively manifest in the region of the O2 (T), it is intriguing to suggest that photoexcitation of the porphyrin close to the thymine C2=O band may influence the local hydration structure of that bond. Indeed, it has been proposed that the CuTMyP4-thymine exciplex may form *via* a bridging H_2_O (or D_2_O) molecule, rather than directly to the C2=O bond as shown in Fig. 1, and that the hydration sphere around the nucleobase has a crucial role in stabilising the exciplex.^31^

The presence of features characteristic of both exciplex and triplet state in the TRIR spectra may reflect the diversity of binding modes available in {d(CGCAAATTTGCG)}_2_, including groove-binding and intercalation. This mirrors previous Raman studies where CuTMPy4 bound to mixed-sequence systems such as poly(dA-dC).poly(dG-dT) was proposed to undergo excited-state relaxation through at least three separate routes.^15^ Nevertheless, the data presented here demonstrates how TRIR can be used to unravel the various DNA interactions of versatile binders such as CuTMPyP4. It may also be noted that previous studies on the perturbation of nucleobase vibrations by photo-excited compounds have focused on intercalators. Compounds such as CuTMPyP4 that can bind in the grooves and may potentially interact with a number of base-pair steps in addition to the DNA spine of hydration, merit further experimental and theoretical investigation.

## Conclusion

The time-resolved infra-red data presented in the paper shows that this technique is a valuable complement to transient absorption as it not only monitors the transient spectroscopic properties of the porphyrin but can concurrently provide information of changes in the biomolecule. Thus, it is possible to use TRIR to detect and distinguish spectroscopically between different types of five-coordinate complexes (previously described as exciplexes) formed by interactions of the CuTMPyP4 upon excitation. In the present case, the TRIR of the 5-coordinate complex produced with D_2_O is quite different in shape from that of the triplet state formed when the porphyrin is intercalated between the base-pairs of {d(GC)_5_}_2_ (in this latter case the absorption band at 1632 cm^−1^ is much stronger than the bleach at 1645 cm^−1^). With thymine-containing nucleic acids the TRIR method also unambiguously demonstrates the binding of the porphyrin to the C2=O carbonyl group of that nucleobase.

## Experimental

Cu(ii)-5,10,15,20-*meso*-tetrakis(*N*-methylpyridinium-4-yl)porphyrin tetrachloride (CuTMPyP4) was purchased from Inochem Ltd and used without further purification. HPLC-purified oligodeoxynucleotide sequences were obtained from Eurogentec. Polydeoxythymidylic acid was purchased from Sigma-Aldrich. TRIR spectra were recorded on the TR^M^PS system,^32^ and ps-TA spectra recorded on the ULTRA^33^ apparatus, at the Rutherford Appleton Laboratories (UK). Further details on transient spectroscopic measurements are provided in the SI.

## Author contributions

PMK, JMK and SJQ designed and carried out the TRIR and TA experimental work. Data were analysed by PMK and DG. IVS and MT set up and optimised the TRIR and TA instruments. PMK, SJQ and JMK drafted the manuscript, which was critically read by all authors.

## Conflicts of interest

None to declare.

## Supplementary Material

CP-028-D5CP04939C-s001

## Data Availability

Data for this article, including time-resolved absorption spectroscopy and time-resolved infra-red spectroscopy datasets, are available from the UCD Research Data Zenodo Community at https://doi.org/10.5281/zenodo.18939777. Supplementary information (SI): additional spectra, kinetics fits and experimental details. See DOI: https://doi.org/10.1039/d5cp04939c.
