# Supplementary material for: Picosecond time-resolved infra-red spectroscopic study of a water-soluble cationic copper-porphyrin with nucleic acids
Source: Phys Chem Chem Phys. 2026 Mar 5;28(17):10467–73. doi: 10.1039/d5cp04939c (PMC12985699; doi:10.1039/d5cp04939c)
Supplement: CP-028-D5CP04939C-s001 [file CP-028-D5CP04939C-s001.pdf]

Supplementary Information (SI) for Physical Chemistry Chemical Physics.
This journal is © the Owner Societies 2026

Supporting Information

Picosecond time-resolved infra-red spectroscopic study of a water-soluble cationic copper-porphyrin with nucleic acids

Páraic M. Keane,[ab] * Daniel Graczyk,[c] Igor V. Sazanovich,[d] Michael Towrie,[d] Susan J. Quinn[c]* and John M. Kelly[a]*

a. *School of Chemistry, Trinity College Dublin, Dublin 2, D02 P3X2, Ireland.*
b. *School of Chemistry, University of Reading. Whiteknights, Reading RG6 6AD, U.K.*
c. *School of Chemistry, University College Dublin, Dublin 4, D04 V1W8, Ireland*
d. *STFC Central Laser Facility, Research Complex at Harwell, Rutherford Appleton Laboratory, Didcot OX11 0QX, U.K.*

keanepa@tcd.ie, jmkelly@tcd.ie, susan.quinn@ucd.ie

Figures and Tables

Fig. S1. Comparative UV/vis spectra (a) CuTMPyP4 and CuTMPyP4-$\{(GC)_5\}_2$ (b) CuTMPyP4-poly(dT) and CuTMPyP4-$\{d(CGCAAATTTGCG)\}_2$

Fig. S2. Kinetic fits to TA of CuTMPyP4 in phosphate-buffered D_2O (a) biexponential transient (b) biexponential bleach (c) monoexponential transient (d) monoexponential bleach. Area under curve calculated from 440 nm to 500 nm (transient) and 410 nm to 440 nm (bleach)

Fig. S3. Kinetic fits to TRIR of CuTMPyP4 in phosphate-buffered D_2O (a) transient at 1632 cm^{-1} (b) bleach at 1643 cm^{-1}

Fig. S4. TRIR spectrum of CuTMPyP4 in phosphate-buffered D_20 at 50 ps delay

Fig. S5. Steady-state spectra of CuTMPyP4 (1.4 µM) in phosphate-buffered (50 mM) H_2O in the presence of $d\{(GC)_6\}_2$ CuTMPyP4:Nucl = 1:100 (a) Luminescence spectra in absence and presence of $d\{(GC)_6\}_2$ (b) UV/vis spectra in absence and presence of $d\{(GC)_6\}_2$ (c) Excitation spectrum of CuTMPyP4-$d\{(GC)_6\}_2$

Fig. S6. FTIR spectra of systems under study (a) CuTMPyP4; CuTMPyP4 in presence of ODNs (b) $\{d(GC)_5\}_2$ (b) poly(dT) (c) $\{d(CGCAAATTTGCG)\}_2$. [CuTMPyP4] = 500 µM, [ODN] = 500 µM (double strand conc).

Fig. S7. Comparative TRIR spectra of (a) CuTMPyP4 in D_2O (b) CuTMPyP4 in presence of poly(dT). Spectra shown at 2 ps and 50 ps. The blue spectrum in (b) is a subtraction 2 ps – 50 ps.

Fig. S8. TRIR spectroscopic changes observed following 400 nm excitation of 0.5 mM CuTMPyP4 in the presence of $\{d(CGCAAATTTGCG)\}_2$ in buffered D_2O, (a) early delays and (b) later delays.

Fig. S9. Recovery of band at 1577 cm^{-1} for 500 µM CuTMPyP4 in presence of $\{d(CGCAAATTTGCG)\}_2$ (1.0 mM per strand, CuTMPyP4:Nucl = 1:24) with monoexponential fit.

Table S1. Kinetic fitting data for TA/TRIR spectra of CuTMPyP4 in D_2O

Experimental

References

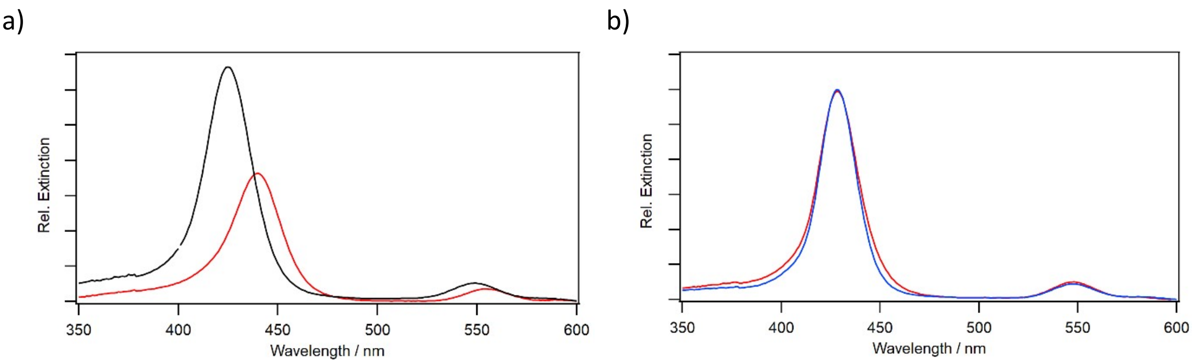

Fig. S1. Comparative UV/vis spectra (a) CuTMPyP4 (black) and CuTMPyP4-{d(GC)$_5$}$_2$ (red) (b) CuTMPyP4-poly(dT) (blue) and CuTMPyP4-{d(CGCAAATTTGCG)}$_2$ (red). [CuTMPyP4] = 500 µM. CuTMPyP4:Nucl = 1:20 (1:24 for CuTMPyP4-{d(CGCAAATTTGCG)}$_2$). 50 µm pathlength in phosphate-buffered D_2O

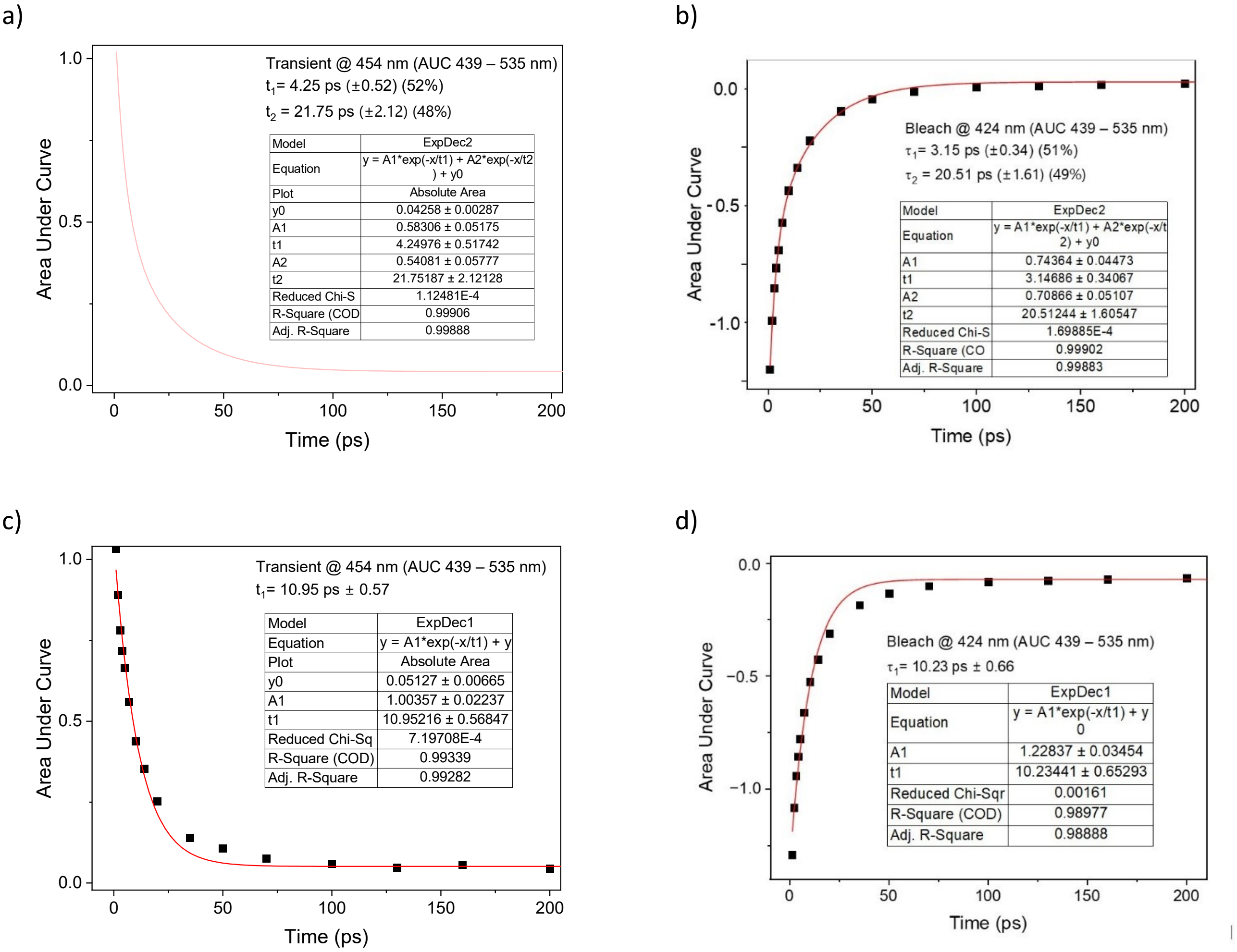

Fig. S2. Kinetic fits to TA of CuTMPyP4 in phosphate-buffered D_2O (a) biexponential transient (b) biexponential bleach (c) monoexponential transient (d) monoexponential bleach. λ_{exc} = 400 nm, 1 µJ. Area under curve calculated from 440 nm to 500 nm (transient) and 410 nm to 440 nm (bleach)

a)

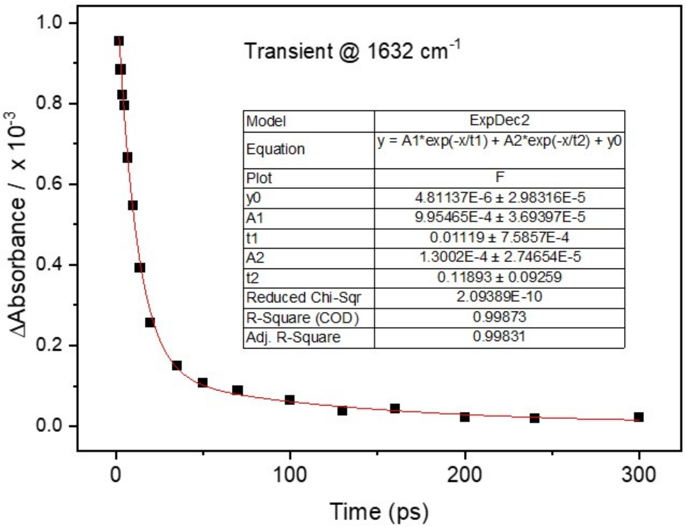

b)

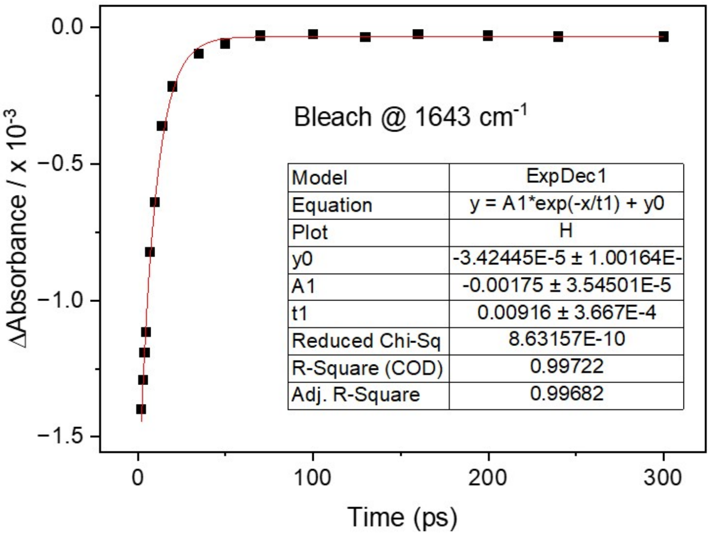

Fig. S3. Kinetic fits to TRIR of CuTMPyP4 in phosphate-buffered D_2O (a) transient at 1632 cm^{-1} (b) bleach at 1643 cm^{-1}. λ_{exc} = 400 nm, 1 μJ.

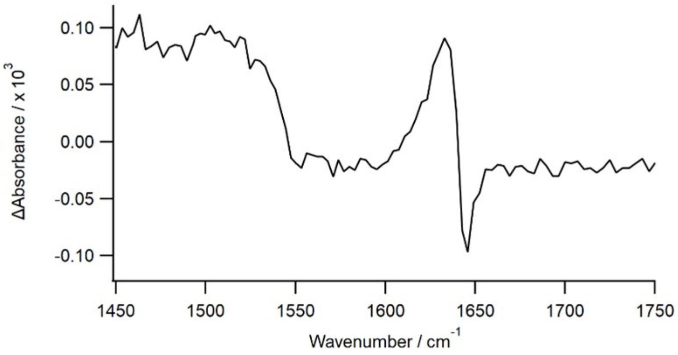

Fig. S4. TRIR spectrum of CuTMPyP4 in phosphate-buffered D_20 at 50 ps delay. The ratio of the transient to bleach areas (1600 cm^{-1} to 1650 cm^{-1} region) is approx. 5 (i.e. >> 1) consistent with formation of the CuTMPyP4 triplet state. λ_{exc} = 400 nm, 1 μJ.

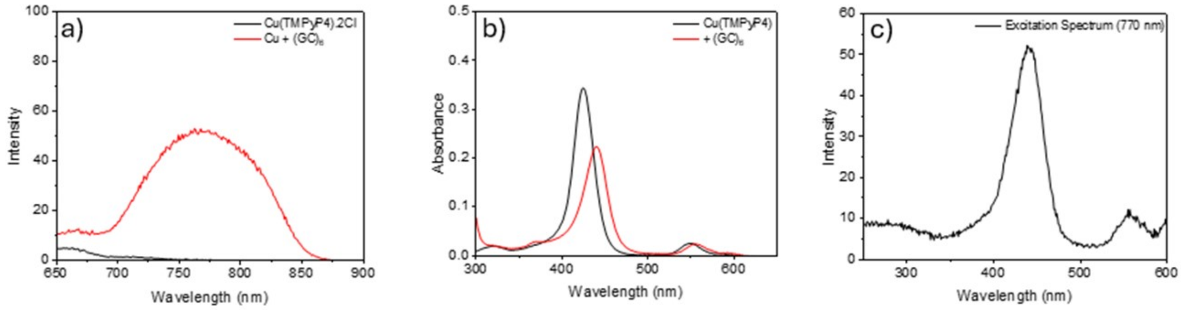

Fig. S5. Steady-state spectra of CuTMPyP4 (1.4 µM) in phosphate-buffered (50 mM) H_2O in the presence of $\{d(GC)_6\}_2$ CuTMPyP4:Nucl = 1:100 (a) Luminescence spectra in absence and presence of $\{d(GC)_6\}_2$ (b) UV/vis spectra in absence and presence of $\{d(GC)_6\}_2$ (c) excitation spectrum of CuTMPyP4-$\{d(GC)_6\}_2$.

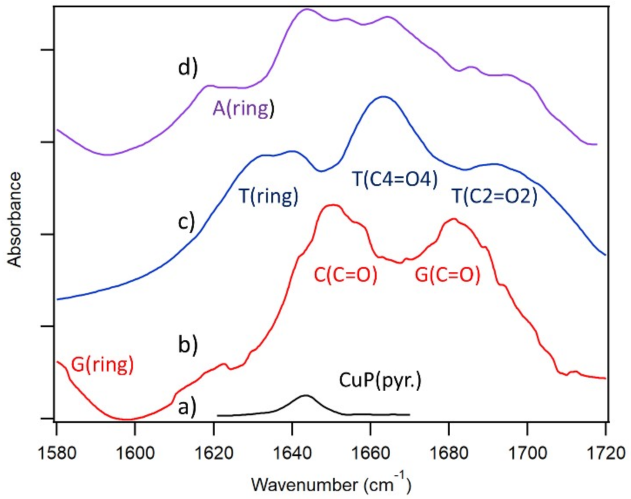

Fig. S6. FTIR spectra of systems under study (a) CuTMPyP4. CuTMPyP4 in presence of ODNs; (b) $\{d(GC)_5\}_2$, red (c) poly(dT), blue (d) $\{d(CGCAAATTTGCG)\}_2$, purple. [CuTMPyP4] = 500 µM. [CuTMPyP4]:[nucleotide] = 1:20 (1:24 for CuTMPyP4-$\{d(CGCAAATTTGCG)\}_2$). 50 µm pathlength in phosphate-buffered D_2O. The significant absorptions are marked on the spectra. Ring vibrations are in-plane stretches of C=C/C=N bonds. Pyr. denotes *N*-methylyridinium modes of CuTMPyP4.

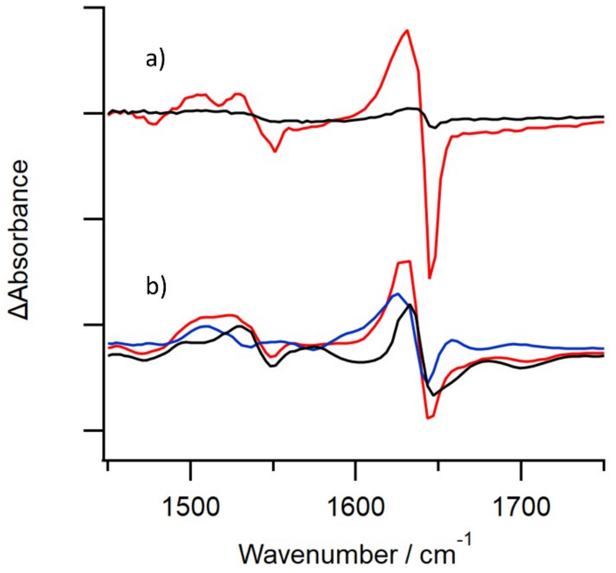

Fig. S7. Comparative TRIR spectra of (a) CuTMPyP4 in phosphate-buffered D_2O (b) CuTMPyP4 in presence of poly(dT). Spectra shown at 2 ps (red) and 50 ps (black). The blue spectrum in (b) is a subtraction 2 ps – 50 ps, representing the species that has decayed over initial 50 ps.

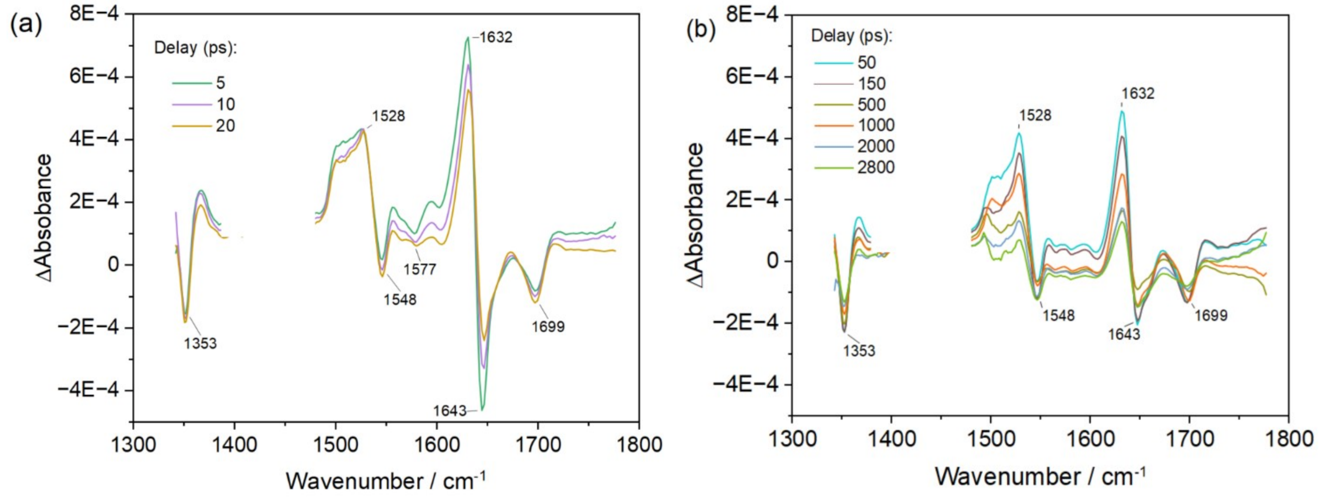

Fig. S8.(a) The early time TRIR spectroscopic changes observed following 400 nm excitation of 0.5 mM CuTMPyP4 in the presence of {d(CGCAAATTTGCG)}$_2$ in buffered D_2O (1.0 mM per strand) showing the depletion of the CuTMPyP4 bands at 1643, 1548, and 1353 cm^{-1}, the latter two features are characteristic of loss of the ground state complex and a significant depletion at 1699 cm^{-1} corresponding to the bleaching of a thymine absorption; (b) the later time changes showing the slow decay of all product bands and recovery of the parent absorptions, the blanked region indicates a region where strong solvent absorptions obscure the spectrum.

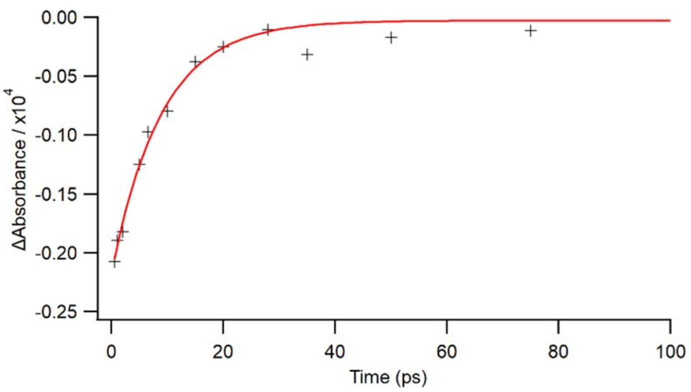

Fig. S9. Recovery of band at 1577 cm^{-1} for 500 µM CuTMPyP4 in presence of {d(CGCAAATTTGCG)}$_2$ (1.0 mM per strand, CuTMPyP4:Nucl = 1:24) with monoexponential fit (τ = 9 ± 1 ps).

Table S1: Kinetic fitting data for TA and TRIR spectra of CuTMPyP4 (50 mM phosphate-buffered D_2O). Fitted using area-under-curve (AUC) and single-point fitting to either monoexponential or biexponential functions.

	**Fitting (are under curve)**	**Monoexp. (ps)**	**Biexp. (ps)**	**Fitting (single point)**	**Monoexp. (ps)**	**Biexp. (ps)**
TA transient	AUC (439 - 535 nm)	11.0 ± 0.6	4.3 ± 0.6 (56%)<br>21.8 ± 2.1 (48%)	460 nm	11.8 ± 0.5	6.3 ± 0.6 (56%)<br>25 ± 2.7 (44%)
TA bleach	AUC (414 – 439 nm)	10.2 ± 0.7	3.2 ± 0.3 (48%)<br>20.5 ± 1.6 (49%)	422 nm	9.7 ± 0.5	4.0 ± 0. 3 (48%)<br>19.7 ± 1.5 (52%)
TRIR transient	AUC (1610 – 1640 cm^{-1})	12.4 ± 0.9	nd	1632 cm^{-1}	13 ± 1	11.2 ± 0.7 (88%)<br>119 ± 9 (12%)
TRIR bleach	AUC (1640 – 1658 cm^{-1})	9.9 ± 0.4	nd	1643 cm^{-1}	9.2 ± 0.4	nd

nd = not determined due to non-convergence or error exceeding fitted lifetime

Experimental

Sample preparation

Samples for time-resolved spectroscopic measurements were prepared by pipetting 50 µL of sample onto 2 mm CaF_2 plates, separated by a 50 µm Teflon spacer, in a liquid demountable cell (Harrick Corp.). Samples were prepared in 99.9% D_2O (Sigma) in 50 mM phosphate buffer (K_2HPO_4/KH_2PO_4, pH = 7, pD =7.4), using $\boldsymbol{\varepsilon}_{424\ nm}$ = 2.31 x 10^5 M^{-1} cm^{-1} for CuTMpyP4.[1]

Data analysis

Random baseline offsets in the spectral data were offset by baseline subtractions post-measurement. Lifetime (τ) analyses of the TA/TRIR data were performed using either area-under-curve or single-point fitting to exponential functions using the Marquardt Levenberg algorithm (IgorPro/OriginPro software)

$$I(t) = y_0 + a_1 e^{-t/\tau_1} + a_2 e^{-t/\tau_2}$$

Picosecond transient absorption (psTA) spectroscopy

Picosecond TA measurements were performed on the ULTRA apparatus at the Central Laser Facility (STFC Rutherford Appleton Laboratories, Harwell, UK).[2] Part of the Thales 10 kHz, 800 nm Ti:Sapphire laser output beam was used to generate a white light continuum (WLC) probe in a CaF_2 plate. The crystal plate was continuously rastered to avoid colour centre formation and to improve pulse-to-pulse stability in the probe. After the sample, the WLC was dispersed through the grating monochromator and detected using a linear silicon array (Quantum Detectors). An appropriate notch filter was placed in front of the monochromator to remove scatter from the excitation beam. The second harmonic radiation at 400 nm provided the pump beam. The polarisation of the pump pulses at the sample was at the magic angle relative to the probe, with an energy of 1 µJ and a spot size ca. 100-150 µm. The instrument response of the setup is estimated to be ca. 200 fs. The spectra were calibrated using a WCT-2065 metal oxide filter. The laser beam was mechanically chopped before the sample at 5 kHz with a C-995 chopper from Terahertz Technologies Inc. Samples were raster scanned in the x and y directions to minimise photodamage and re-excitation effects. Samples were checked before and after the experiment by UV/vis spectroscopy.

Time resolved infrared (TRIR) spectroscopy

The experiments were carried out in the Time Resolved Multiple Probe Spectroscopy, $TR^{M}PS$, mode of the ULTRA instrument.[3] The mid IR probe was generated by difference frequency conversion of the signal and idler from an optical parametric amplifier (TOPAS, Light Conversion) pumped by 4 W of the output of a 10 kHz, 40 fs nm Thales titanium sapphire laser and the 400 nm pump by second harmonic of a 1 kHz, 120 fs, 800 nm Spitfire titanium sapphire laser. The 373 nm pump beam, where used, was provided by a second OPA (TOPAS, Light Conversion) pumped with the fundamental beam of the 10 kHz titanium sapphire laser. The polarisation of the pump pulses at the sample was at the magic angle relative to the probe, with an energy of 1 µJ and a spot size ca. 100-150 µm. The instrument response of the setup is estimated to be ca. 200 fs. By operating in $TR^{M}PS$ mode TRIR spectra spanning picosecond to microsecond timescales were produced. The methods used to obtain microsecond delays are described in detail elsewhere; the systems under investigation in the current study were characterised in the picosecond/nanosecond timescale only.

References

1. R. F. Pasternack and M. A. Cobb, *J. Inorg. Nucl. Chem.*, 1973, **35**, 4327-4339.

2. G. M. Greetham, P. Burgos, Q. Cao, I. P. Clark, P. S. Codd, R. C. Farrow, M. W. George, M. Kogimtzis, P. Matousek, A. W. Parker, M. R. Pollard, D. A. Robinson, Z. J. Xin and M. Towrie, *Appl. Spectrosc.*, 2010, **64**, 1311–1319.

3. G. M. Greetham, D. Sole, I. P. Clark, A. W. Parker, M. R. Pollard and M. Towrie, *Rev. Sci. Instrum.*, 2012, **83**, 103107.
